# On the Influence of the Human Instinct in the Prevention and Cure of Disease, Chiefly in Reference to Diet

**Published:** 1855-06

**Authors:** Thomas Hunt


### ON THE INFLUENCE OF THE HUMAN INSTINCT IN THE PREVENTION AND CURE OF DISEASE, CHIEFLY IN REFERENCE TO DIET.

By Thomas Hunt, Esq., F.R.C.S.

[Read at the Medical Society of London (Physiological Meeting), April 9th, 1855.]

The term *instinct* has been used by naturalists and physiologists in two significations. It has been applied by some to those involuntary organic movements common to animals and plants, which are the result of stimuli applied to a structure possessed of irritability,—such as the respiratory and circulatory actions, the intestinal movements, the opening and closing of the petals of plants, the collapse of the leaves of the mimosæ, &c. By other writers, the term instinct has been used in a more restricted sense, as applicable only to those voluntary movements of animals which are not the result of reasoning, but of sensation only, and which, though normally conducive to the welfare of the animal or his race, are not directed to any intelligible end of which the animal is conscious. Popular writers have thoughtlessly restricted this function to the lower animals, and supposed it to supply the place of reason; whereas animals are not destitute of reason, nor is man himself without his instincts. Adopting the definition of that clear and elegant writer, Dr. Paley, namely, that "instinct is a propensity prior to experience and independent of instruction, and tending to the preservation of the individual or his race",—I venture to assert that, in this sense of the term, it is by no means true that man has no instincts, any more than it is true that the lower animals are destitute of reason.

Every infant comes into the world endowed with a propensity to suck and to swallow; a propensity prior to experience and independent of instruction; and this propensity has a tendency to preserve the life and health of the child, as well as that of the mother. Thus early in life is exhibited the influence of the human instinct in the prevention of disease. As the teeth advance, the propensity to suck is gradually supplanted by an instinctive tendency to bite and masticate; and the craving for solid food, and especially for meat, suggests, better than any reasoning on the subject, what kind of diet will now be the most conducive to health. By and bye, reason dawns; and some would conclude that instinct, being no longer necessary, ceases to exert its influence: but this is a mistake. Instinct presides over the whole physical man from the cradle to the grave, suggesting the kind of diet most needful to the individual, and best adapted to his habits of life, whether they be active or indolent, laborious or sedentary,—whether consisting chiefly of mental, or almost entirely of bodily exertion. In mature life its suggestions tend most powerfully to the preservation of the individual and his race; and in old age the active and aspiring propensities of youth and early manhood succumb to the sobriety, caution, and discretion, which are necessary for the preservation of the enfeebled frame, and the lengthening out of the span of life.

It is not my present purpose to inquire into the nature and tendency of all the human instincts (a subject, nevertheless, of the highest physiological interest), but to confine my observations to those instincts alone which are connected with hygiene, or the preservation and restoration of health; and it may be comprised under two heads, *regimen* and *diet*. A few words only on regimen.

By *regimen* I mean clothing, bathing, air, exercise bodily and mental, recreation, sleep, and all essentials to health, exclusive of diet and medicine: and I maintain that if all of these were left to be regulated by the instinctive dictates of natural desire or choice, they would, under this influence, be rendered more conducive to health, than when submitted wholly to the artificial restrictions of science. Pure country air is admitted to be better adapted for respiration than the polluted atmosphere of cities, or the fetor of badly drained habitations: and how much more readily and willingly do men seek the recreations of the country, under the influence of natural desire, than they pursue them in obedience to those rules of science, which are not only imperfect and erring in themselves, but little appreciated by the million. The love of simple pleasures, which find their home among flowers and forests, mountains and rivers, and the wide sea, is far more strong, not only in childhood but at all ages, in uncontaminated minds than the love of pleasures and amusements artificially invented. So, with regard to dress, the heat of summer, not less than the cold of winter, instinctively suggests clothing suited to the season, and far better adapted to our wants than the habits of fashion or the devices of art. Active exercise, again, is as agreeable to the youthful and vigorous, as is quiet to the aged, and rest to the weak and weary. And activity of mind, which is so essential to the health of the body, is the result of a natural propensity. The mind most ill at ease, most given to hypochondriasis, most fretful and melancholy, is that which has no occupation, no alternations of hope and fear, no incentives of ambition or adventure. The longing for a sailor's life is the instinct of active youth. And even the cares of a family, the natural result of following the suggestions of the conjugal instinct (for the sexual instinct of man, and even of other mammals, is monogamous in its natural dictates), are more agreeable, as well as more salutary, than the gloom and selfishness of celibacy. "If matrimony," said Dr. Johnson, "has many cares, celibacy has no pleasures." Indeed, it is difficult to point out any mode of life which has a tendency to preserve health, which is not also a natural habitude of our race, suggested to our choice by pure instinct.

I propose, however, on this occasion to restrict my observations to *diet*, as dictated by natural desire, in health and disease.

*Diet.*—It is not disputed, that a properly regulated diet tends both to preserve health, and to restore it when impaired; or, in other words, to prevent disease and to cure it. But, to prescribe for a given case such a diet as shall best prevent or cure disease, is by no means so easy a problem as popular belief supposes. The voice of antiquity, indeed, agrees with modern science in commending temperance. But instinct is beforehand even here. Young infants and children are naturally temperate. Gluttony is not the vice of childhood, but of adult age: and the reason why children eat too much occasionally, is that they are always longing for proper and natural, but *forbidden* food: and when they can get it they eat it voraciously, on the principle of making hay while the sun shines; and thus they eat more than their instinct dictates, being guided more by perverted reason than by actual hunger. Instinct, left to itself, is always temperate.

Again, it is generally believed that the most wholesome diet is that which is *simple* and *nutritious:* and so far as this is true, it is also the dictate of nature. But these terms are both of them general and undefined; and there are many peculiarities of temperament in which they are not equally applicable to the diet which is salutary, and some in which they are not applicable at all. He must be blinded indeed by theory who does not perceive that there are many individuals who do *not* thrive on a simple diet, but require a great variety and complicity of food to keep them in good health. And it is even doubtful whether this be not true of a large majority of persons in a highly civilized state of society. Then again, the term *nutritious* has its limits. Some persons require (for reasons not known to us) full twice the quantity of nutriment which suffices for the health and strength of others; and while a mixture of innutritious with nutritious matter is essential to all, the proportions in which these should be combined can only be ascertained by consulting the instinctive desire of each. But few can thrive long on bread and meat alone. Some will prefer rice, potatoes, or other vegetables, in large proportions. And with all due respect for those reformers who would lure mankind to temperance by the doctrines of teetotalism, we must contend that there are some persons, the aged especially, who cannot be kept in health without a diet in some degree alcoholic and stimulating: and it is idle to deny that in them the desire for a moderate allowance is a dictate of nature. Nor must we overlook the strong desire for acids which some individuals experience especially at certain times. In others a desire for alkalies or neutral salt is equally strong. There are indeed a hundred peculiarities of idiosyncrasy under which the appetite elects, and the system requires, a corresponding peculiarity of diet. Thus what is one man's necessary food is literally another's man's poison; and it is absolutely impossible to discover, except by the instinct of the individual, what is the kind of food required, either in health or disease. I have therefore yet to learn on what principles medical practitioners presume, as a matter of course, to interfere with the tastes and appetites of the patient. Modern chemistry, with all its mighty achievements, microscope in hand, sheds but a very feeble light on this intricate subject, and stands helpless and dumb-foundered by the side of the little child, who knows what is best fitted for the wants of its mystic economy, far better than the most searching analysis of the secretions and excretions can disclose. I will go a step further, and say that the great majority of cases of functional dyspepsia, which one meets with in practice in these days of temperance, actually originate in, or at least are much aggravated by, a too rigid adherence to artificial rules of diet, a too restricted use of the good things which nature has provided, and a too strict avoidance of certain palatable articles of diet, such as fruits, acids, sweets, fresh vegetables, vinous liquors, etc. And in practice I have always found that dyspepsia, when unconnected with organic disease, is best treated by the patient avoiding intemperance on the one hand, and, on the other hand, repudiating every one of the rules of diet which he has been induced to adopt, or which may have been handed down from generation to generation.

That these rules are always conceived in ignorance and adopted without due reflection, will appear evident if we only call to mind the very limited knowledge we possess of the chemistry of digestion. If the human system were unendowed with life, if the stomach were a simple alembic, and if the various processes of digestion, assimilation, secretion and excretion, absorption and deposit were simply chemical changes, which might be effected in the laboratory as easily as in an organized vital structure, then, indeed, we might examine the secretions, and presume to correct them by a prescribed and restricted diet. Modern chemistry would then triumph over every difficulty of this kind, and, guided by careful analysis, might undertake, without fear of disappointment, the task of synthetically providing for all the wants and wastes of the human system. Even the conditions of disorder and disease might in that case ultimately become indicative of some special diet and regimen adapted for each particular case. But, unfortunately, the chemistry of life—that vital, hidden process by which our food is converted into a living fluid, and this fluid into bone and muscle and ligament and medulla—is governed by laws of which we know practically absolutely nothing, and the very existence of which we only infer from very distant and obscure analogy. Here and there we get a ray of light by the analysis of vital products and experiments on living animals; but of the synthesis of these products, as of the precise materials required for its elaboratlon, we are utterly and perhaps hopelessly ignorant.

Are we not then guilty of temerity when we presume to dictate to the economy of digestion what materials are best suited to its purpose? We are indignant at the conduct of the ignorant, unqualified practitioner of medicine, because that he, knowing nothing of anatomy or the principles of medicine, yet prescribes recklessly and in the dark in cases where the aid of an enlightened physician is at hand. But if we, being wholly ignorant of what the case requires, and having no means at hand of discovering what is wanted, except the light which the patient's instinct throws on the question, do yet, regardless of that light, prescribe blindly for our patient a particular diet suggested only by routine,—wherein are we better than the quack? "The greater part of a philosopher's life," says the eloquent Dugald Stewart, "must be spent in unlearning the errors of the crowd." And the first thing we have to unlearn here is this assumed knowledge of the science of diatetics. When we have learned that we know, practically, absolutely nothing, we shall then have taken the first step towards learning something.

Happily, where science and reason fail, nature has provided, in the gift of instinct, an unerring guide. Physiologists have remarked, that the act of respiration is too important an affair to be trusted to the volition of so capricious a creature as man. If it were in the power of so fickle a being to suspend his breathing whenever he might feel for a moment oppressed with *ennui*, his life would never be safe. And doubtless the essential question of aliment is too important to be left to the chances of discovery by science. Generations of men were nourished before chemistry was dreamed of. And the mere circumstance of man having an infinite variety of food within his reach, including a host of poisonous herbs, must have created the necessity of a provision to insure a selection of those natural productions of which, without endangering his life, he might freely eat. That provision has been given: and it consists of a supply of nerves, which are the seat of sensations fully competent to guide his choice, the food which is palatable being alone wholesome, and, *vice versâ*, that which is repulsive being unwholesome. Equally at a loss should we have been, not only with respect to the quality, but also as regards the quantity of food required to sustain us in health, whether an ounce or a pound; as well as the frequency of our necessary repasts, whether they should be partaken of once in an hour, once in a day, or once in a week. Who taught us when to eat and when to drink, and when to cease eating and drinking? To the natural sensations of hunger and thirst are committed this care over our dietetic well-being. Science would have been equally at fault here: instinct alone is our guide. The palate directs us to the kind of food; the appetite to the quantity required by the system. This knowledge is confined to our own wants; the preposterous conceit that we can prescribe for the wants of others by any other rule than their own desires, must have originated in some empirical fraud practised by astrologers in the dark ages; and there is not a more remarkable circumstance in the history of human science, than that so fallacious a conceit should have been handed down to this enlightened age, when almost every similar phantom has fled before the advancing light of inductive science.

Now, if it be so extremely difficult to determine, *à priori*, by the aid of chemistry and physiology alone, what kind of diet will best subserve the wants of the body when in health, I maintain, *à fortiori*, that it is, if possible, still more difficult in disease. And it is my firm conviction, that our most judicious prescriptions in the way of medicine are frequently countervailed and rendered nugatory by our perverseness in presuming to regulate the diet by arbitrary rules.

But this leads us to inquire whether nature has provided us with a dietetic instinct in disease, as well as in health. I think both analogy and experience must determine the question in the affirmative. The carnivorous animal, when out of health, seeks the healing influence of a vegetable diet, and eats grass like an ox. In like manner when man is in a state of early fever, his appetites are widely diverse from those of health. His thirst frequently becomes urgent if not insatiable, while the very sight and smell of food is nauseating to him; or if any appetite for food remain, he prefers acid fruits and succulent vegetables to dry solid food. And who shall presume to say that this is wrong, or that the salutary instinct should be thwarted? As the fever advances and takes on new types, the longings of the patient vary accordingly: wine, acids, strange mixtures, or articles nominally improper and indigestible, are craved for, and if he can only get them, he often dates his recovery from his extravagant indulgence of this apparently capricious taste. Indeed, in no case is it more important that a strange longing of this nature should be indulged than at the crisis of a fever. A young lady, suffering from malignant typhus of an alarming nature, became livid and nearly pulseless, her mouth and teeth were covered with black sordes,—there was the glazed eye, the short breathing, the subsultus tendinum, the picking and pinching of the bed-clothes, and the Hippocratic face, which are too well known as the harbingers of death. She faintly asked for some pickled walnuts, and was allowed to indulge her longing freely. She ate nine large walnuts, and drank a pint of the spiced vinegar in which they were pickled. The skin became moist, the pulse returned, she slept soundly, awoke refreshed, and rapidly became convalescent. In Asiatic cholera, the instinctive desire for cold drinks, and often for acid drinks, is almost always present, and few will recover under any treatment unless it is freely indulged. Two patients in collapse, who were abandoned to their fate, drank each many quarts of cider, and both recovered. One of them had a second attack some months afterwards. He begged earnestly for cider, but it was forbidden him, as a most improper thing; and—he died! Many of the most distressing cases of dyspepsia may be relieved, and most of them cured, by allowing the patient (who is often worn down by a rigid diet) an unlimited indulgence in every variety of food which a capricious appetite may suggest. I say this advisedly, after thirty years unflinching trial of the plan. The difficulty consists in convincing the patient that he may freely abandon himself to his appetites. Living by rule has become a habit with him, which it is really difficult to throw off entirely, and yet unless it *be* entirely repudiated, he does not give nature fair play, and he may consequently remain a dyspeptic still: for nature abhors restrictions most of all in these very cases, and a slight deviation from her dictates is often visited with heavy penalties. And I may here observe, that a great variety of food is generally preferred by patients of weak digestion, and when it is so preferred it will be found *most salutary*. A patient who could not digest a mutton chop, although and because he had been ordered to live on nothing else, recovered the tone of his digestive organs by indulging in a great variety of French dishes, of which he was particularly fond. One day he had, however, a severe attack of spasms in the chest and sick headache, which he attributed to having taken some animal jelly for lunch. I asked him if he was particularly fond of this article of food. He replied he had no great relish for it, but he liked to look at it. I said, "Then look at it, but do not eat it, for you cannot enjoy the sight of it when you have once swallowed it: look at that which is beautiful, but only swallow what is *palatable*". He took the hint, and having long acted upon it, has now recovered from all his dyspeptic miseries, and is a healthy man.

The longings of patients are sometimes perfectly unaccountable. A diarrhœa, which persisted while the diet of the patient consisted of boiled rice and mutton, yielded so soon as he was permitted to indulge freely in pickled onions and cider, for which he had expressed a longing desire. A young barrister was taken ill on the midland circuit, complained of great lassitude, and could take nothing but lemonade. This stopped the vomiting, and relieved him much. He consulted a physician, who forbad the beverage, and prescribed a gentle aperient and an effervescing neutral saline. The next day the sickness was worse, the abhorrence of food had increased, and a sense of extreme languor and fatigue came over him. He returned to town, and consulted me on his arrival at home. The longing for lemonade still haunted him, but he had most religiously abstained. I ordered the juice of two lemons to be squeezed into a tumbler of cold water, and sweetened according to his taste. He drank the whole of it, and expressed himself immediately relieved of all his symptoms. He now thought he could eat a few native oysters. He swallowed a dozen of them whole and alone. The next day he was nearly well, but weak; he drank lemonade *ad libitum*, and became convalescent without any other medicine except a gentle aperient. He took the juice of three lemons daily. Facts, strange as these, will multiply before us, if we will only wait and watch, and allow nature to have fair play. And we should be cautious not to class such facts as anomalies. There is simply some deterioration of the circulating fluids, which is to be corrected by indulgence in those instinctive dictates, which both in health and disease supply the still existing lack of therapeutical knowledge.

I trust enough has been advanced to prove that the instincts of human nature, if fairly and confidently trusted, would exercise a most salutary influence in the prevention and cure of disease, and that the appetites of the patient will supply a rule of diet infinitely more trustworthy than any other rule, and especially than the maxims which too often guide our practice.

It only remains to be observed that this rule, like all other physical laws, has its exceptions, which exceptions, when rightly considered, will be found rather to prove the rule than to render it nugatory.

Among the afflictions to which the human system is subject, none are more common in the present day than those which may be included under the general term *malæsthesia,* consisting of disordered or morbid sensations, often depending upon no obvious cause. The affections termed *neuralgic* are of this kind: and this name is given when the sensations are *painfully* disordered. But it often happens that the natural sensation of a part is so strangely altered as to convey an erroneous impression to the mind. The nerves convey a false impulse and tell an untrue tale. Patients describe this variously, but generally as though the part itself were wrong. A limb (for instance) feels "numbed", or "swollen", or "wrung", or "dragged", or "heavy", or "hot", or "cold", or "prickly", when there is really nothing the matter with it; no swelling, no change of temperature, or weight, or appearance. There is simply some disordered function in the nerves of the part or their *origin,* or some reflex morbid action, which conveys a false impression. Now, in like manner, the sensations of the palate or of the stomach may become morbidly deranged, and a desire for unnatural food, or for an unnatural quantity, may usurp the dominion of the natural instinct for a time. Thus the chlorotic young female longs for chalk, or cinders, or shavings, or sawdust: things in fact that cannot easily be swallowed, much less digested:—so, in the habitual drunkard, and occasionally in the temperate and sober, there exists an unnatural and morbid craving for spirituous liquors, the indulgence of which is obviously destructive of the health. Now and then patients tell us that they are extremely fond of lobsters, or of veal, or soles, or walnuts, or chesnuts; but that these things always make them ill. Most of these cases consist of morbid sensations, and form exceptions to the rule; and here reason and experience step in, and we have no practical difficulty in defining the limits to which instinct may be trusted, or, in other words, we can easily distinguish between a natural appetite and a morbid craving. But the longing is not always to be called morbid merely because the thing desired apparently disagrees with the patient. He may long for something which excites vomiting or diarrhœa. these very disorders proving salutary in the end.

I cannot conclude without alluding to some exceptions to the rule that instinct dictates the quantity as well as the quality of our aliments. It cannot be doubted that modern cookery may, by setting before us a succession of spiced and savoury food, tempt the appetite beyond the wants of the system: but here the appetite becomes artificially excited, and for the time morbid, not instinctive. Again, in health, instinct may suggest correctly the necessity for *some* vinous stimulant, but not the quantity required: this must be left to the dictates of reason. A sense of languor, sinking, or distress at the præcordia, or of weariness and faintness, is the indication for wine; and under a small dose, the painful sensation is relieved, and an agreeable sense of comfort is imparted. This is the time to stop; if we go on until a sense of exhilaration is excited, the desire is no longer instinctive; and reason, which ought to decide the question, is very apt also not to be in very vigorous exercise. The question, therefore, of the quantity of stimulus required is a very delicate one; and he is wise who errs on the side of temperance.

In these and in all other cases in which instinct fails (and we know it fails when the very articles most desired evidently injure the health)—in all these cases science steps in. But it is not chemical or physiological science, it is only *empirical.* We discover errors in diet by the individual experience of the case in hand, not by the principles of medicine, which would certainly fail us. And the common-sense of the patient, if he be duly observant of his own experience, will tell him, far better than the physician, what will most disagree with him. Individuals differ so much in this respect from each other, that there is no rule but the results of individual experience. Happily, however, it is extremely rare that the natural preferences *are* erroneous. Science may err widely, experience may be misinterpreted and misunderstood: but if there be any dictates which deserve to be called *unerring* as a general rule, they are the dictates of instinct and not of science. For science has its limits; and he is the best philosopher who can discern those limits, and confess his ignorance of what lies beyond them.

